# Postgraduate gastroenterology training and continuing medical education in Africa: challenges, opportunities, and future directions

**DOI:** 10.1097/MS9.0000000000004075

**Published:** 2025-10-13

**Authors:** Hareesha Rishab Bharadwaj, Aditya Gaur, Khabab Abbasher Hussien Mohamed Ahmed, Dushyant Singh Dahiya

**Affiliations:** a Faculty of Biology Medicine and Health, The University of Manchester, Manchester, UK; b Yeovil District Hospital, Somerset NHS Foundation Trust, Higher Kingston, Yeovil, UK; c Faculty of Medicine, University of Khartoum, Khartoum, Sudan; d Division of Gastroenterology, Hepatology and Motility, The University of Kansas School of Medicine, Kansas, USA

**Keywords:** Africa, Capacity building, Endoscopy, Gastroenterology training, Medical education, Workforce development

## Abstract

Africa faces a critical shortage of gastroenterologists amidst a growing burden of digestive diseases. Despite an alarming rise in gastrointestinal (GI) conditions – including peptic ulcers, GI cancers, and infectious colitis – postgraduate training in gastroenterology remains underdeveloped. This narrative review examines the structural, educational, and systemic challenges facing GI training across the continent, drawing from peer-reviewed literature, institutional surveys, and global health databases. Findings reveal substantial heterogeneity in training program length, curriculum standards, and procedural exposure. Advanced techniques like endoscopic retrograde cholangiopancreatograph and endoscopic ultrasound remain inaccessible to most trainees, while simulation facilities and didactic teaching are often limited. Faculty shortages, lack of protected research time, and minimal access to conferences further compromise academic development and contribute to workforce attrition. Despite these challenges, several innovative approaches offer hope. Low-cost simulation models, tele-education, and hybrid conference formats are improving access to training. Regional centers of excellence and North–South collaborations – such as partnerships between African institutions and the British Society of Gastroenterology (BSG) – have demonstrated success in building local capacity. The expansion of digital platforms, diaspora engagement, and public-private partnerships presents further opportunities. Recommendations include implementing competency-based assessments, supporting faculty development, creating regional accreditation bodies, and investing in infrastructure. Addressing systemic inequities in conference access and leveraging geospatial surveillance tools could also enhance disease mapping and policy planning. Ultimately, strengthening gastroenterology education in Africa requires coordinated regional and international action to develop sustainable, context-sensitive training ecosystems. This review provides a roadmap for achieving equitable specialist training and improving digestive health outcomes across the continent.

## Introduction

Africa faces a severe shortage of gastroenterological expertise, despite the rising burden of both infectious and noncommunicable digestive diseases^[1–3]^. The prevalence of upper gastrointestinal (GI) conditions, such as peptic ulcers and esophageal malignancy-related dysphagia, is notably high in regions like Senegal, while lower GI conditions – including hemorrhoids, infectious colitis, and colorectal cancers – are also becoming more widespread^[3–5]^. The increasing incidence of GI malignancies has led to significant morbidity, diminished quality of life, financial strain, and high mortality rates. A recent global burden of disease (GBD) analysis identified Africa as the region with the highest disability-adjusted life years (DALYs) due to digestive diseases, showing the least improvement over the past decade^[6,7]^. Nations such as Egypt and the Central African Republic recorded the highest DALY rates, while southern sub-Saharan Africa bore the greatest proportional burden of upper digestive tract diseases^[7]^. Future projections indicate a substantial increase in GI cancer cases across Africa by 2030^[8]^.

**HIGHLIGHTS**

- Africa faces a critical shortage of gastroenterologists amid a growing burden of digestive diseases like gastrointestinal (GI) cancers, peptic ulcers, and infectious colitis.
- Postgraduate training programs are highly variable, with limited access to advanced procedures (e.g., endoscopic retrograde cholangiopancreatograph and endoscopic ultrasound), faculty shortages, and minimal research infrastructure.
- Emerging innovations – such as low-cost simulation models, tele-education, regional centers of excellence, and international partnerships – are helping bridge key training gaps.
- The review recommends implementing competency-based assessments, investing in infrastructure, promoting equitable conference access, and using geospatial tools to inform policy and improve GI health outcomes across the continent.

Despite this escalating need, GI training and service capacity remain grossly inadequate. The availability of gastroenterologists in the region is significantly below required standards, with certain nations such as Nigeria having only 60 gastroenterologists for a nation of over 100 million inhabitants^[9]^. Furthermore, significant disparities exist in access to formal training, with diagnostic colonoscopy available in only 14% of surveyed nations^[10]^, and fewer than half of practitioners receive structured academic training^[11]^. A pan-European survey reported that 70.1% of trainees performed fewer than 50 endoscopic retrograde cholangiopancreatographies (ERCPs) annually, with only 5.0% of trainers in the same category. Further surveys reveal that African trainees frequently reported feeling underprepared in key areas, including hepatology (19%), inflammatory bowel disease (17%), GI bleeding (15%), and infectious gastroenterology, particularly HIV-related conditions (13%)^[12]^. These findings underscore the urgent need for a prompt, structured, and standardized discussion on the current state of GI postgraduate training in the region, as well as resource-appropriate training initiatives to address competency gaps and strengthen gastroenterology education across the continent.

## Methodology

This article employs a narrative review methodology to explore the current landscape of gastroenterology and hepatology training in Africa. The objective was to examine the structural, procedural, and systemic challenges faced by training programs across the continent, while highlighting successful initiatives and proposing actionable strategies for reform. To achieve this, data were drawn from a combination of peer-reviewed literature, global health databases, institutional reports, and gray literature.

The literature search was conducted using PubMed, Google Scholar, and the World Health Organization’s (WHO) Global Health Observatory. Sources published between 2000 and 2025 were considered to ensure both historical and contemporary coverage. Search terms included combinations of “gastroenterology training,” “Africa,” “endoscopy education,” “health workforce,” “low- and middle-income countries,” “ERCP,” “EUS,” “simulation training,” “conference access,” and “medical education Africa.” Emphasis was placed on multicountry analyses, regional surveys, and authoritative position statements from bodies such as the European Society of Gastrointestinal Endoscopy (ESGE), the World Gastroenterology Organization (WGO), and national gastroenterology societies. Additional data from the GBD project and institutional audits were used to contextualize disease burden and infrastructure gaps.

In line with the TITAN AI reporting framework^[13]^, artificial intelligence (AI) was used solely to enhance the clarity, grammar, and linguistic presentation of the manuscript. Generative AI (ChatGPT, OpenAI GPT-4, 2024) was employed during the writing and revision stages to refine human-authored text. The tool was not used for developing research questions, conducting the literature search, performing any data analysis, or interpreting results. The authors take full responsibility for the content, accuracy, and integrity of all AI-assisted sections.

The AI tool used was ChatGPT (OpenAI, GPT-4, accessed via chat.openai.com in July 2025). No fine-tuning, plug-ins, or advanced parameters (e.g., temperature or token manipulation) were applied; interactions were prompt-based using default settings. The system operated through a cloud-based API and was not integrated with any institutional databases or external software.

Only text written by the authors was entered into the AI platform for linguistic refinement. No patient data, protected health information, or confidential material were provided. All inputs were compliant with General Data Protection Regulation (GDPR)/ Health Insurance Portability and Accountability Act (HIPAA) standards. No institutional approvals were required, as no identifiable data were shared.

All AI-modified text was reviewed by the first and senior authors for factual accuracy, consistency of meaning, and clinical appropriateness. Outputs were cross-referenced with the original human-authored drafts. Where AI suggestions were unclear or inappropriate, they were either revised manually or discarded. The authors acknowledge the limitations of AI in capturing nuanced, context-specific language and ensured human oversight was maintained throughout the process.

To mitigate the risk of algorithmic bias and ensure ethical compliance, AI-generated revisions were assessed to avoid erasure or distortion of content related to underrepresented populations or settings. No conflicts of interest or financial relationships with AI vendors exist. The use of AI in this project adhered to relevant ethical standards and journal requirements.

Prompts used for refinement included general phrasing such as “Improve grammar and clarity of the following paragraph” or “Rephrase this text for academic tone.” No AI-generated content is archived for independent replication, as its use was limited to stylistic enhancement and did not influence the scientific content, interpretation, or conclusions.

## The current landscape of gastroenterology and hepatology postgraduate education in Africa

The structure of postgraduate gastroenterology training in Africa varies significantly across countries and institutions. An analysis of 38 training programs in 14 African nations highlights substantial heterogeneity in both content and duration. While 44.7% of programs span 2 years, others last 3 years (23.7%) or up to 6 years (15.8%), reflecting the lack of regional standardization. Entry requirements also differ: 42.1% of programs require both written and oral assessments, whereas 15.8% lack a transparent selection process. Curriculum design is equally varied – 47.1% of programs use locally adapted curricula, while 20.6% align with international standards such as the Accreditation Council for Graduate Medical Education. Subspecialty training remains limited, with hepatology (31.6%) and advanced endoscopy (26.3%) being the most commonly available fellowships. Financial constraints are pervasive, with 78.1% of programs relying solely on government funding and only 6.3% accessing research grants^[13]^.

Another major barrier to educational consistency is the shortage of trained faculty. Nearly 90% of programs operate with five or fewer consultant gastroenterologists, resulting in high trainee-to-faculty ratios. This significantly impacts supervision quality and limits structured clinical teaching – only 26.4% of trainees receive more than 35 weeks of instruction annually. Didactic learning is similarly sparse; 37.4% of trainees attend fewer than 5 weeks of formal lectures per year. Compounding these issues, fewer than 30% of programs have access to simulation equipment, restricting hands-on skill development outside of clinical environments. Additionally, heavy clinical workloads are common, with 74.6% of trainees working over 40 hours per week^[13]^.

Despite these limitations, some programs have implemented short-term initiatives to bolster training. Gastroenterology symposia, such as one held in Zambia, have provided valuable learning opportunities across borders. This event attracted trainees from several African countries, with 55.5% seeking to improve general gastroenterology knowledge and 13.8% aiming to enhance endoscopic skills. International collaborations – especially those involving UK-based experts – have also created specialized educational opportunities, reinforcing the value of external partnerships^[14]^. Research engagement remains modest due to infrastructural and financial constraints. Around 40% of trainees dedicate fewer than 5 hours per month to research, often conducted in their personal time due to a lack of protected time or funding. This fragmented engagement is exacerbated by the limited availability of research grants and mentorship opportunities^[15]^. Still, localized efforts have demonstrated that impactful research is possible within resource-limited settings^[16–19]^.

Academic development is further hindered by infrastructural inadequacies. The ESGE survey (2018) found that only half of the 15 African countries surveyed have formal endoscopy training centers. In 8 of the 13 countries assessed, more than 90% of endoscopists reported receiving training confined to basic procedures. Many hospitals lack essential diagnostic equipment – such as proctoscopes – and advanced tools like fluoroscopy and automatic disinfection systems are largely absent^[13,20]^. In Nigeria, for example, just two public hospitals provide ERCP services despite having over 200 endoscopy centers serving a population of 200 million^[21]^.

This severe underexposure to advanced procedures such as ERCPs and endoscopic ultrasound (EUS) remains a critical issue. While 84.9% of programs set a minimum threshold for esophagogastroduodenoscopy (EGD), and 81.8% mandate colonoscopy training, only 59.3% of trainees perform more than 40 colonoscopies. For advanced procedures, the situation is more dire – 74 and 86% of trainees report no exposure to ERCP and EUS, respectively^[11]^. The lack of procedural access compromises the development of essential skills required for independent practice, such as EGDs, and calls for the implementation of competency-based learning models across programs.

These systemic challenges contribute significantly to poor workforce retention. Approximately 40% of gastroenterology graduates emigrate within 5 years, driven by limited career development, research opportunities, and unsatisfactory working conditions. The ESGE survey further underscores the severity of this issue, reporting physician densities ranging from 14 to 1192 per million in Africa, starkly lower than the 2554 per million observed in the United States. As a result, waiting times for emergency endoscopy can extend from days to weeks, highlighting the strain on already overstretched systems^[22]^.

Conferences and regional networking platforms play a pivotal role in mitigating these challenges by facilitating knowledge exchange and skill development. However, African trainees often face financial and logistical barriers to participation. Although regional gatherings like the Society for Gastroenterology and Hepatology in Nigeria and the South African Gastroenterology Society offer relevant content, institutional funding constraints frequently limit attendance. Global conferences such as Digestive Disease Week and United European Gastroenterology Week provide exposure to international standards but remain largely inaccessible due to travel costs, registration fees, and visa issues^[16–19]^.

## Future prospects

Simulation-based learning – including low-cost models such as EASIE-R – presents a promising solution to address current gaps in ultrasound and procedural training. Complementing this, digital platforms have expanded access: virtual didactics now comprise more than half of lectures in 16.5% of African gastroenterology programs. To ensure consistency in skill acquisition, competency-based assessments could standardize procedural benchmarks, guaranteeing that all trainees across Africa attain the competencies needed for independent clinical practice. However, the full potential of these modern educational modalities depends on broader structural reforms, including faculty development: “Train the Trainer” programs can enhance supervision quality, especially given that 90% of academic gastroenterology divisions offer only 10–20% protected time for faculty career development and research, while 10% offer little or none^[23]^.

Creating centers of excellence in strategic locations – such as South Africa and Egypt – would magnify training capacity by centralizing advanced procedural instruction, competency verification, and faculty exchanges. These hubs could serve as regional focal points for mentorship, research, and evaluation, thereby addressing disparities in skills and access^[24,25]^. Building on this concept, key retention strategies – such as loan-forgiveness, structured academic career pathways, and diaspora engagement – must be prioritized. These initiatives could align with WHO Agenda 2063 workforce targets, support the formation of a pan-African gastroenterology accreditation body, and foster public-private partnerships to sustain growth^[26]^.

Positive examples already exist: Sudan’s successful partnership with the BSG and South Yorkshire Endoscopy Group has trained multidisciplinary teams through hands-on workshops and fellowships^[27]^. Sudan’s Khartoum WGO Training Center, launched in 2015, is another model – combining training, research, and regional outreach. In Tanzania, the Gastroenterology Foundation e.V.’s Dar es Salaam Endoscopy and Oncology Training Center has trained 10 young doctors to date, offering state-recognized certification over a 2-year program and plans to expand advanced procedures like ERCP. Senegal offers a compelling South–South model: between 2005 and 2009, a Belgian–Senegalese project established a therapeutic endoscopy suite at a university hospital in Dakar, leading to trainee autonomy in basic therapeutic endoscopy and increasing therapeutic activity from 0 to 12% within 2 years of funding completion^[28]^.

Tele-education and telemedicine offer further promise. In Uganda, a remote mentorship system connected international trainers to trainees in Kyabirwa, enabling effective remote supervision of endoscopic procedures and expanding to the African Esophageal Cancer Consortium^[29]^. Remote training has proven feasible with minimal cost and has been commended for enabling flexibility across time zones. Additionally, the Pan-African e-Network Project – implemented between India and the African Union – has established satellite-based tele-education and tele-medicine networks in over 30 countries, offering a scalable model for remote gastroenterology education and mentorship. These initiatives demonstrate that technology-enabled training can reach rural and underserved areas, providing resilience during political instability or infrastructure gaps.

Investment in equipment and infrastructure remains critical. Sustainable partnerships with manufacturers – such as BSG-facilitated equipment donations or leasing – could deploy mobile endoscopy units to underserved rural areas^[27]^. Training local biomedical engineers to maintain this equipment would ensure service continuity; this was a notable need in contexts like Sudan. Public-private partnerships could also support regional training hubs, mirroring Sudan’s model in Khartoum.

Knowledge-sharing initiatives fostered through regional collaboration should be scaled. North–South collaborations – linking sub-Saharan African countries like Sudan and Nigeria with North African nations like Egypt and Tunisia – can enhance skill and resource transfer. Conferences, such as Sudanese Society of Gastroenterology meetings attracting over 400 attendees, exemplify scalable models for broader participation^[30]^. Beyond education, research advocacy is essential: disease burden data on GI cancers and ulcer prevalence should guide policy and investment, with organizations like BSG lobbying WHO and African Union support – especially in postconflict settings^[31]^. Efforts to engage the African diaspora through short-term training and remote mentorship can bolster specialist development. Deployable endoscopy clinics and mobile units – already piloted in other low-income countries – have potential in conflict, resource-limited regions and those with infection epidemics. Here, telemedicine consultations can ensure continuity of specialist care^[32–35]^.

Access to conferences remains a barrier. Trainees from Low- and Middle-Income Countries (LMICs) pay disproportionately high registration costs – often $268 on average – which represents a significant share of annual income ($2000–$9400), and only 19% of conferences offer reduced fees. Conference participation suffers further due to inadequate virtual access, with only 28.6% offering virtual attendance and fewer than 60% discounted rates, while restrictive visa policies and travel costs compound the issue. To remedy this, advocacy for 50–80% discounts, fee waivers for delegates from conflict zones (e.g., Sudan and Democratic Republic of the Congo (DRC), increased hosting in Africa (e.g., South Africa, Egypt, Nigeria, and Kenya), and expanded hybrid models with on-demand recordings are essential. Support from organizations like BSG and WGO via travel grants, as well as pharmaceutical sponsorship and dedicated presentation slots for African researchers, will help bridge equity gaps and reinforce mentorship pathways^[15,36–39]^.

Drawing on approaches used in mapping infectious diseases such as malaria and visceral leishmaniasis, incorporating geospatial surveillance and climate-sensitive analytics could enhance our understanding of GI disease patterns across Africa^[40,41]^. These methodologies could inform targeted interventions, particularly in regions facing concurrent epidemiological and environmental pressures^[40,41]^. Together, these combined strategies – simulation, digital learning, regional hubs, tele-education, diaspora engagement, equipment investment, and equitable conference participation – offer a robust roadmap for advancing gastroenterology training in Africa. Continued expansion and replication of successful models will drive sustainable progress across the continent.

## Conclusion

Transforming African gastroenterology training requires a multipronged approach that addresses systemic resource limitations, curricular standardization, and workforce retention. While current data reveal significant gaps in procedural exposure – particularly for ERCP and EUS – as well as faculty support, emerging solutions in simulation training, digital education, and regional collaboration present viable pathways forward. Through coordinated action by national societies, international partners, and policymakers, African nations can develop sustainable training ecosystems capable of addressing the continent’s unique GI needs. Future research should evaluate the implementation and outcomes of these proposed interventions to guide ongoing quality improvement efforts.

## Data Availability

Data availability is not applicable to this article as no new data were created or analyzed in this study.

## Research registration unique identifying number (UIN)

No UIN exists as this study is an editorial.

## Suggested reviewers

1.Prof. Athanasios Alexio, Department of Science and Engineering, Novel Global Community Educational Foundation, Hebersham, NSW 2770, Australia. E-mail: alexiou@ngcef.net.

2. Prof. Vladyslav Sikora, Department of Clinical and Experimental Medicine, University of Foggia, Via Napoli, 20, Foggia 71122, Italy. E-mail: V.sikora@med.sumdu.edu.ua
